# Supplementary material for: Free-IgE as a Predictor of Responsiveness to Omalizumab in Oral Corticosteroid-Dependent Asthma Patients
Source: Int J Mol Sci. 2025 Mar 21;26(7):2852. doi: 10.3390/ijms26072852 (PMC11989120; doi:10.3390/ijms26072852)

Supplementary materials

Table S1. Longitudinal measures for the study cohort.

Variable	Visit					
	0	1	2	3	4	5
Total IgE, IU/mL	285 (280)	777 (854)	630 (618)	565 (488)	546 (458)	514 (446)
FVC, %	77.4 (20.1)	80.7 (17.2)	73.8 (16.4)	76.4 (16.7)	76.9 (19.0)	75.9 (16.5)
FEV_1, %	63.5 (20.5)	66.0 (18.3)	61.6 (18.6)	64.4 (20.0)	65.0 (20.3)	66.0 (21.1)
FEV_1/FVC ratio, %	62.9 (14.2)	63.6 (15.2)	62.7 (14.2)	63.1 (13.9)	64.1 (14.6)	64.7 (13.8)
Dose of omalizumab, mg	-	298 (114)	288 (120)	256 (116)	269 (134)	244 (160)
Dose of corticosteroids, mg	5.7 (5.1)	-	-	-	-	2.3 (4.0)
Free IgE, IU/mL	-	6.6 (14.4)	-	-	-	-
Free-to-total IgE ratio, %	2.33 (3.74)	0.88 (1.71)	1.10 (2.34)	1.09 (1.73)	1.12 (1.74)	1.09 (1.51)
FEV_1 criterion						
FEV_1 follow-up ≤ basal (non-responder)	-	10 (32.3%)	19 (61.3%)	16 (51.6%)	13 (41.9%)	14 (45.2%)
FEV_1 follow-up > basal (responder)	-	21 (67.7%)	12 (38.7%)	15 (48.4%)	18 (58.1%)	17 (54.8%)
Dose of corticosteroids criterion						
Dose follow-up ≥ basal (non-responder)	-	-	-	-	-	15 (48.4%)
Dose follow-up < basal (responder)	-	-	-	-	-	16 (51.6%)
Dose of omalizumab criterion						
Dose follow-up ≥ basal (non-responder)	-	31 (100%)	29 (93.5%)	23 (74.2%)	23 (74.2%)	20 (64.5%)
Dose follow-up < basal (responder)	-	0 (0%)	2 (6.45%)	8 (25.8%)	8 (25.8%)	11 (35.5%)
Omalizumab response						
Non-responder	-	-	-	-	-	7 (22.6%)
Responder	-	-	-	-	-	24 (77.4%)

Values are presented as mean (standard deviation) for continuous variables and frequency (percentage) for categorical variables. FEV_1, forced expiratory volume in 1 second; FVC, forced vital capacity; IgE, immunoglobulin E; IU, international unit.

Table S2. Measures by treatment response at visit 5 (three criteria).

Variable	Non-responder N=7	Responder N=24	p-value
Women	4 (57.1%)	17 (70.8%)	0.658
Age at visit 0, years	54.9 (18.5)	50.8 (16.8)	0.614
Weight, kg	72.4 (10.1)	73.1 (19.8)	0.901
Height, cm	161 (9.8)	160 (9.9)	0.707
BMI, kg/m^2	27.9 (4.05)	28.5 (6.52)	0.782
Total IgE at visit 0, IU/mL	228 (144)	302 (309)	0.383
FVC at visit 0, %	86.6 (19.5)	74.7 (19.9)	0.188
FEV_1 at visit 0, %	74.1 (17.0)	60.4 (20.6)	0.099
FEV_1/FVC ratio at visit 0, %	66.4 (17.2)	61.8 (13.5)	0.533
Dose of corticosteroids at visit 0, mg	3.14 (6.09)	6.50 (4.69)	0.214
Total IgE at visit 1, IU/mL	539 (339)	847 (948)	0.196
FVC at visit 1, %	82.7 (9.7)	80.1 (19.0)	0.633
FEV_1 at visit 1, %	70.3 (12.5)	64.8 (19.7)	0.389
FEV_1/FVC ratio at visit 1, %	64.9 (15.6)	63.2 (15.4)	0.810
Dose of omalizumab at visit 1, mg	289 (80)	300 (123)	0.789
Total IgE at visit 2, IU/mL	547 (342)	655 (681)	0.577
FVC at visit 2, %	73.0 (10.9)	74.0 (17.9)	0.858
FEV_1 at visit 2, %	58.0 (15.7)	62.6 (19.6)	0.529
FEV_1/FVC ratio at visit 2, %	59.7 (17.2)	63.6 (13.5)	0.598
Dose of omalizumab at visit 2, mg	289 (80)	288 (130)	0.965
Total IgE at visit 3, IU/mL	499 (324)	585 (531)	0.606
FVC at visit 3, %	71.4 (7.4)	77.8 (18.5)	0.184
FEV_1 at visit 3, %	57.3 (14.9)	66.5 (21.1)	0.215
FEV_1/FVC ratio at visit 3, %	60.0 (17.0)	64.0 (13.2)	0.580
Dose of omalizumab at visit 3, mg	268 (121)	253 (117)	0.782
Total IgE at visit 4, IU/mL	679 (596)	507 (417)	0.497
FVC at visit 4, %	67.7 (19.1)	79.5 (18.5)	0.179
FEV_1 at visit 4, %	53.6 (16.2)	68.4 (20.4)	0.068
FEV_1/FVC ratio at visit 4, %	60.6 (16.6)	65.1 (14.1)	0.526
Dose of omalizumab at visit 4, mg	364 (170)	241 (111)	0.109
Total IgE at visit 5, IU/mL	563 (417)	500 (462)	0.739
FVC at visit 5, %	70.9 (9.6)	77.3 (17.9)	0.224
FEV_1 at visit 5, %	58.3 (17.6)	68.3 (21.9)	0.236
FEV_1/FVC ratio at visit 5, %	61.6 (18.0)	65.6 (12.6)	0.592
Dose of omalizumab at visit 5, mg	364 (170)	209 (142)	0.057

Variable	Non-responder	Responder	p-value
	N=7	*N=24*	
Dose of corticosteroids at visit 5, mg	3.14 (6.09)	2.08 (3.31)	0.672
Free IgE at visit 1, IU/mL	3.21 (4.98)	7.62 (16.1)	0.254
Free-to-total IgE ratio at visit 0, %	1.60 (1.93)	2.55 (4.13)	0.403
Free-to-total IgE ratio at visit 1, %	0.73 (0.87)	0.93 (1.90)	0.700
Free-to-total IgE ratio at visit 2, %	0.78 (0.94)	1.20 (2.62)	0.514
Free-to-total IgE ratio at visit 3, %	1.02 (1.33)	1.11 (1.85)	0.898
Free-to-total IgE ratio at visit 4, %	0.74 (0.91)	1.23 (1.92)	0.354
Free-to-total IgE ratio at visit 5, %	0.78 (0.91)	1.18 (1.65)	0.412
FEV_1 criterion at visit 1			0.658
FEV_1 follow-up ≤ basal	3 (42.9%)	7 (29.2%)	
FEV_1 follow-up > basal	4 (57.1%)	17 (70.8%)	
FEV_1 criterion at visit 2			0.200
FEV_1 follow-up ≤ basal	6 (85.7%)	13 (54.2%)	
FEV_1 follow-up > basal	1 (14.3%)	11 (45.8%)	
FEV_1 criterion at visit 3			0.004
FEV_1 follow-up ≤ basal	7 (100%)	9 (37.5%)	
FEV_1 follow-up > basal	0 (0%)	15 (62.5%)	
FEV_1 criterion at visit 4			0.105
FEV_1 follow-up ≤ basal	5 (71.4%)	8 (33.3%)	
FEV_1 follow-up > basal	2 (28.6%)	16 (66.7%)	
FEV_1 criterion at visit 5			0.002
FEV_1 follow-up ≤ basal	7 (100%)	7 (29.2%)	
FEV_1 follow-up > basal	0 (0%)	17 (70.8%)	
Dose of corticosteroids criterion at visit 5			0.002
Dose of corticosteroids follow-up ≥ basal	7 (100%)	8 (33.3%)	
Dose of corticosteroids follow-up < basal	0 (0%)	16 (66.7%)	
Dose of omalizumab criterion at visit 1			Not estimable
Dose of omalizumab follow-up ≥ basal	7 (100%)	24 (100%)	
Dose of omalizumab follow-up < basal	0 (0%)	0 (0%)	
Dose of omalizumab criterion at visit 2			>0.999
Dose of omalizumab follow-up ≥ basal	7 (100%)	22 (91.7%)	
Dose of omalizumab follow-up < basal	0 (0%)	2 (8.33%)	
Dose of omalizumab criterion at visit 3			>0.999
Dose of omalizumab follow-up ≥ basal	5 (71.4%)	18 (75.0%)	
Dose of omalizumab follow-up < basal	2 (28.6%)	6 (25.0%)	
Dose of omalizumab criterion at visit 4			0.138
Dose of omalizumab follow-up ≥ basal	7 (100%)	16 (66.7%)	

Variable	Non-responder	Responder	p-value
	N=7	*N=24*	
Dose of omalizumab follow-up < basal	0 (0%)	8 (33.3%)	
Dose of omalizumab criterion at visit 5			0.072
Dose of omalizumab follow-up ≥ basal	7 (100%)	13 (54.2%)	
Dose of omalizumab follow-up < basal	0 (0%)	11 (45.8%)	

Values are presented as mean (standard deviation) for continuous variables and frequency (percentage) for categorical variables. FEV_1, forced expiratory volume in 1 second; FVC, forced vital capacity; IgE, immunoglobulin E; IU, international unit.

Table S3. Measures by treatment response at visit 5 (omalizumab criterion).

Variable	Dose of omalizumab follow-up ≥ basal	Dose of omalizumab follow-up < basal	p-value
	N=20	*N=11*	
Women	14 (70.0%)	7 (63.6%)	>0.999
Age at visit 0, years	52.2 (15.4)	50.9 (20.3)	0.860
Weight, kg	74.4 (19.8)	70.3 (14.3)	0.504
Height, cm	161 (7.7)	159 (13.1)	0.730
BMI, kg/m2	28.6 (6.36)	27.8 (5.53)	0.719
Total IgE at visit 0, IU/mL	271 (256)	310 (330)	0.739
FVC at visit 0, %	79.5 (23.4)	73.5 (12.4)	0.355
FEV_1 at visit 0, %	64.8 (22.5)	61.0 (17.0)	0.596
FEV_1/FVC ratio at visit 0, %	61.9 (13.0)	64.6 (16.8)	0.645
Dose of corticosteroids at visit 0, mg	4.7 (4.6)	7.6 (5.8)	0.164
Total IgE at visit 1, IU/mL	695 (799)	926 (969)	0.510
FVC at visit 1, %	82.3 (18.8)	77.8 (14.2)	0.462
FEV_1 at visit 1, %	65.2 (18.7)	67.6 (18.2)	0.722
FEV_1/FVC ratio at visit 1, %	61.3 (13.3)	67.7 (18.1)	0.316
Dose of omalizumab at visit 1, mg	278 (73.4)	334 (162)	0.293
Total IgE at visit 2, IU/mL	630 (718)	631 (404)	0.996
FVC at visit 2, %	73.7 (15.1)	73.9 (19.4)	0.976
FEV_1 at visit 2, %	59.0 (16.7)	66.2 (21.7)	0.358
FEV_1/FVC ratio at visit 2, %	60.5 (13.7)	66.6 (14.8)	0.275
Dose of omalizumab at visit 2, mg	270 (78.5)	320 (171)	0.373
Total IgE at visit 3, IU/mL	598 (574)	507 (286)	0.562
FVC at visit 3, %	75.4 (14.4)	78.1 (21.0)	0.715
FEV_1 at visit 3, %	62.9 (18.3)	67.2 (23.6)	0.608
FEV_1/FVC ratio at visit 3, %	62.4 (13.8)	64.5 (14.7)	0.701

Variable	Dose of omalizumab follow-up ≥ basal	Dose of omalizumab follow-up < basal	p-value
	N=20	*N=11*	
Dose of omalizumab at visit 3, mg	285 (116)	205 (101)	0.056
Total IgE at visit 4, IU/mL	600 (540)	447 (241)	0.285
FVC at visit 4, %	74.9 (18.5)	80.5 (20.1)	0.459
FEV_1 at visit 4, %	62.0 (18.5)	70.6 (22.9)	0.296
FEV_1/FVC ratio at visit 4, %	63.1 (14.5)	65.9 (15.3)	0.623
Dose of omalizumab at visit 4, mg	319 (137)	177 (60.7)	<0.001
Total IgE at visit 5, IU/mL	589 (522)	378 (219)	0.127
FVC at visit 5, %	75.3 (15.5)	76.8 (19.0)	0.829
FEV_1 at visit 5, %	64.4 (20.6)	68.9 (22.8)	0.596
FEV_1/FVC ratio at visit 5, %	63.7 (14.3)	66.5 (13.1)	0.582
Dose of omalizumab at visit 5, mg	319 (137)	109 (97.0)	<0.001
Dose of corticosteroids at visit 5, mg	3.10 (4.7)	0.91 (1.6)	0.071
Free IgE at visit 1, IU/mL	8.06 (17.0)	4.01 (7.78)	0.373
Free-to-total IgE ratio at visit 0, %	2.63 (4.39)	1.79 (2.20)	0.486
Free-to-total IgE ratio at visit 1, %	1.17 (2.09)	0.37 (0.27)	0.109
Free-to-total IgE ratio at visit 2, %	1.42 (2.87)	0.53 (0.50)	0.187
Free-to-total IgE ratio at visit 3, %	1.30 (2.07)	0.70 (0.73)	0.257
Free-to-total IgE ratio at visit 4, %	1.27 (2.07)	0.83 (0.89)	0.418
Free-to-total IgE ratio at visit 5, %	1.17 (1.76)	0.94 (0.96)	0.632
FEV_1 criterion at visit 1			0.711
FEV_1 follow-up ≤ basal	7 (35.0%)	3 (27.3%)	
FEV_1 follow-up > basal	13 (65.0%)	8 (72.7%)	
FEV_1 criterion at visit 2			0.708
FEV_1 follow-up ≤ basal	13 (65.0%)	6 (54.5%)	
FEV_1 follow-up > basal	7 (35.0%)	5 (45.5%)	
FEV_1 criterion at visit 3			0.376
FEV_1 follow-up ≤ basal	12 (60.0%)	4 (36.4%)	
FEV_1 follow-up > basal	8 (40.0%)	7 (63.6%)	
FEV_1 criterion at visit 4			0.721
FEV_1 follow-up ≤ basal	9 (45.0%)	4 (36.4%)	
FEV_1 follow-up > basal	11 (55.0%)	7 (63.6%)	
FEV_1 criterion at visit 5			0.709
FEV_1 follow-up ≤ basal	10 (50.0%)	4 (36.4%)	
FEV_1 follow-up > basal	10 (50.0%)	7 (63.6%)	
Dose of corticosteroids criterion at visit 5			0.034
Dose of corticosteroids follow-up ≥ basal	13 (65.0%)	2 (18.2%)	

Variable	Dose of omalizumab follow-up ≥ basal	Dose of omalizumab follow-up < basal	p-value
	N=20	*N=11*	
Dose of corticosteroids follow-up < basal	7 (35.0%)	9 (81.8%)	
Dose of omalizumab criterion at visit 1			Not estimable
Dose of omalizumab follow-up ≥ basal	20 (100%)	11 (100%)	
Dose of omalizumab follow-up < basal	0 (0%)	0 (0%)	
Dose of omalizumab criterion at visit 2			>0.999
Dose of omalizumab follow-up ≥ basal	19 (95.0%)	10 (90.9%)	
Dose of omalizumab follow-up < basal	1 (5.0%)	1 (9.1%)	
Dose of omalizumab criterion at visit 3			0.009
Dose of omalizumab follow-up ≥ basal	18 (90.0%)	5 (45.5%)	
Dose of omalizumab follow-up < basal	2 (10.0%)	6 (54.5%)	
Dose of omalizumab criterion at visit 4			<0.001
Dose of omalizumab follow-up ≥ basal	20 (100%)	3 (27.3%)	
Dose of omalizumab follow-up < basal	0 (0%)	8 (72.7%)	
Omalizumab response at at visit 5			0.072
Non-responder	7 (35.0%)	0 (0%)	
Responder	13 (65.0%)	11 (100%)	

Values are presented as mean (standard deviation) for continuous variables and frequency (percentage) for categorical variables. FEV_1, forced expiratory volume in 1 second; FVC, forced vital capacity; IgE, immunoglobulin E; IU, international unit.

Table S4: Descriptive and differences (final - basal) of disease control measures for responders, according to the 3-criteria at visit 5.

	**Baseline** N = 24	**V5** N = 24	**Difference (final - basal)** [1]	**95% CI** [1]	**p-value** [1]
**FEV1 [%]**			**7.9**	**(2.4, 13)**	**0.007**
Mean (SD)	60.38 (20.64)	68.29 (21.86)			
Median (P25, P75)	61 (45, 77)	69 (55, 82)			
Min, Max	18, 94	20, 106			
**Dose of corticosteroids [mg]**			**-4.4**	**(-6.6, -2.2)**	**<0.001**
Mean (SD)	6.50 (4.69)	2.08 (3.31)			
Median (P25, P75)	8 (4, 8)	0 (0, 4)			
Min, Max	0, 16	0, 12			
**Dose of omalizumab [mg]**			**-91**	**(-165, -17)**	**0.019**
Mean (SD)	300.00 (123.14)	209.38 (141.58)			

Median (P25, P75)	300 (300, 300)	150 (150, 300)			
Min, Max	150, 750	0, 600			
**Total IgE [IU/mL]**			**198**	**(51, 346)**	**0.011**
Mean (SD)	301.70 (308.61)	499.86 (461.89)			
Median (P25, P75)	184 (105, 364)	405 (199, 621)			
Min, Max	37, 1,175	61, 1,948			

[1] Paired t-test.
CI, confidence interval; P25, 25% percentile; P75, 75% percentile; SD, standard deviation.

Table S5: Descriptive and differences (final - basal) of disease control measures for responders, according to the reduction of omalizumab dose criterion at visit 5.

	Baseline N = 11	V5 N = 11	Difference (final - basal) [1]	95% CI [1]	p-value [1]
**FEV_1 [%]**			**7.9**	**(-1.4, 17)**	**0.088**
Mean (SD)	61.00 (16.96)	68.91 (22.79)			
Median (P25, P75)	61 (49, 75)	81 (48, 88)			
Min, Max	35, 86	34, 92			
**Dose of corticosteroids [mg]**			**-6.7**	**(-11, -2.9)**	**0.003**
Mean (SD)	7.64 (5.78)	0.91 (1.64)			
Median (P25, P75)	8 (4, 12)	0 (0, 1)			
Min, Max	0, 16	0, 4			
**Dose of omalizumab [mg]**			**-225**	**(-333, -117)**	**<0.001**
Mean (SD)	334.09 (162.12)	109.09 (97.00)			
Median (P25, P75)	300 (300, 338)	150 (0, 150)			
Min, Max	150, 750	0, 300			
**Total IgE [IU/mL]**			**68**	**(-128, 264)**	**0.458**
Mean (SD)	310.10 (330.39)	378.09 (218.60)			
Median (P25, P75)	170 (107, 391)	297 (231, 519)			
Min, Max	37, 1,152	98, 790			

[1] Paired t-test.
CI, confidence interval; P25, 25% percentile; P75, 75% percentile; SD, standard deviation.

Figure S1. Evolution of different variables according to clinical response (three criteria responders vs non-responders). Values are presented as mean (95% confidence interval).

Subfigure A: Evolution of FVC; Subfigure B: Evolution of FEV_1; Subfigure C: Evolution of IgE; Subfigure D: Evolution of omalizumab dose

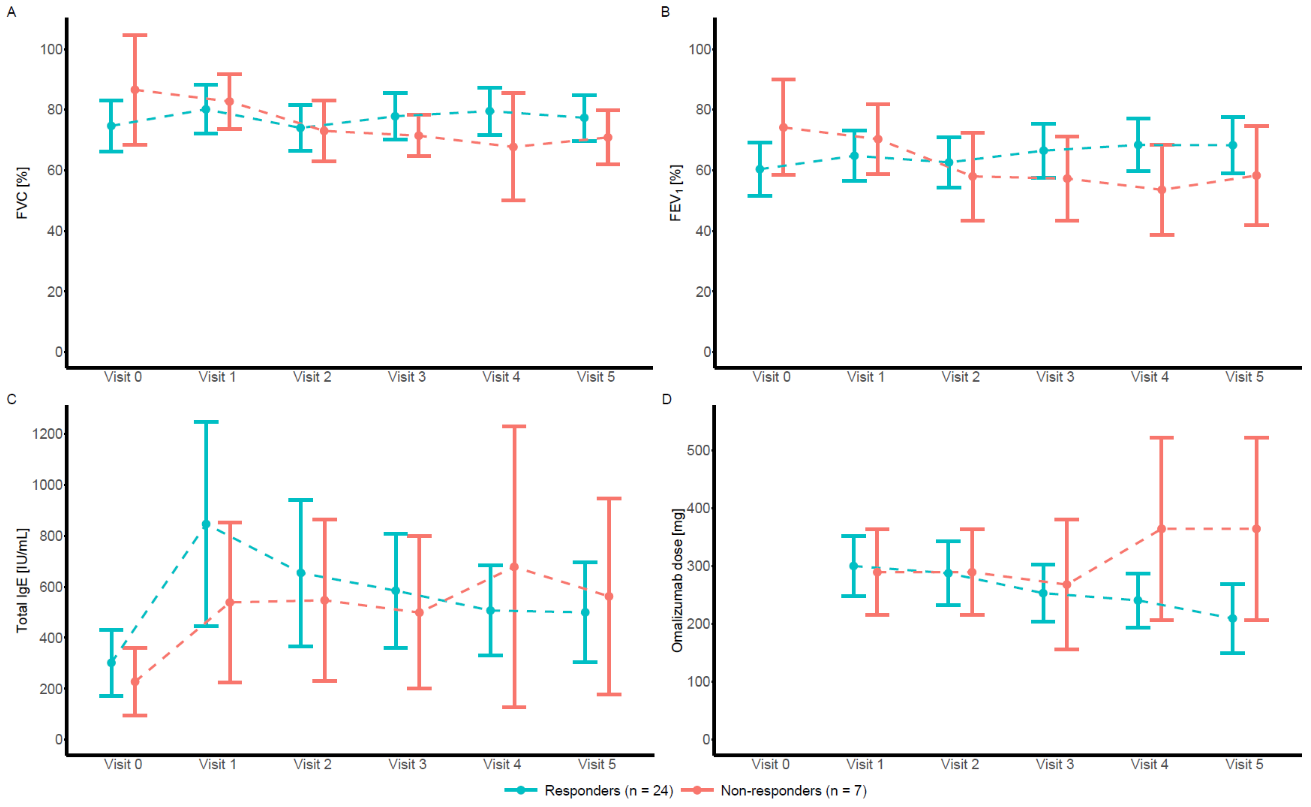

Supplement: Supplementary file 1 [file ijms-26-02852-s001.zip › ijms-3149270-supplementary.pdf]
